# Functional brain changes in sarcopenia: evidence for differential central neural mechanisms in dynapenic older women

**DOI:** 10.1007/s40520-023-02391-1

**Published:** 2023-04-08

**Authors:** Wiebke Trost, Mélany Hars, Natalia Fernandez, François Herrmann, Thierry Chevalley, Serge Ferrari, Gabriel Gold, René Rizzoli, Patrik Vuilleumier, Andrea Trombetti

**Affiliations:** 1 Division of Bone Diseases, Department of Medicine, Faculty of Medicine, Geneva University Hospitals, Rue Gabrielle-Perret-Gentil 4, 1205 Geneva 14, Switzerland; 2 Laboratory for Behavioural Neurology and Imaging of Cognition, Campus Biotech, University of Geneva, Geneva, Switzerland; 3 Division of Geriatrics and Rehabilitation, Department of Rehabilitation and Geriatrics, Faculty of Medicine, University Hospitals of Geneva, Geneva, Switzerland

**Keywords:** Sarcopenia, Brain, fMRI, Older people

## Abstract

**Background:**

The European Working Group on Sarcopenia in Older People (EWGSOP2) recently revised its definition and diagnostic criteria for sarcopenia, placing muscle strength at the forefront. The pathogenesis of dynapenia (or low muscle strength) is still not fully understood, but there is emerging evidence that central neural factors constitute critical determinants.

**Methods:**

Our cross-sectional study included 59 community-dwelling older women (mean age 73.1 ± 4.9 years). Participants underwent detailed skeletal muscle assessments for muscle strength defined by handgrip strength and chair rise time measurements using the recently published EWGSOP2 cut-off points. Functional magnetic resonance imaging (fMRI) was assessed during the performance of a cognitive dual-task paradigm, consisting of a baseline, two single-tasks (motor and arithmetic) and one dual-task (motor and arithmetic combined).

**Results:**

Forty-seven percent (28/59) of participants were classified as dynapenic. fMRI results revealed a differential recruitment of motor circuits in the brain during the dual-task condition in dynapenic as compared with non-dynapenic participants. In particular, while the brain activity during the single-tasks did not differ between the two groups, only during the dual-task non-dynapenic participants showed significant increased activation in dorsolateral prefrontal and premotor cortex, and in supplementary motor area as compared to dynapenic participants.

**Conclusion:**

Our results point to a dysfunctional involvement of brain networks associated with motor control in dynapenia in a multi-tasking paradigm. A better knowledge of the link between dynapenia and brain functions could provide new impulses in the diagnosis and interventions for sarcopenia.

**Supplementary Information:**

The online version contains supplementary material available at 10.1007/s40520-023-02391-1.

## Introduction

Sarcopenia represents a geriatric syndrome which is characterized as a progressive skeletal muscle disease that is associated with a wide range of negative health outcomes including mobility disability, falls, fractures, frailty, hospitalisations and mortality [1, 2]. Recently, the European Working Group on Sarcopenia in Older People revised its definition for sarcopenia, suggesting new classification criteria (EWGSOP2) [3]. Although the focus in sarcopenia has previously been on muscle mass, there is a recent shift towards a framework emphasizing the role of low muscle strength, known as dynapenia, for the definition of sarcopenia. Research over the past decade has highlighted that measures of muscle strength better predict poor health outcomes than measures of muscle mass [4].

While the symptoms of dynapenia have thus far been well characterized, important gaps in knowledge remain concerning its etiology and pathogenesis. It has long been thought that muscle mass loss was the main contributor of low muscle strength. But it is now acknowledged that this age related decline in muscle strength might also be due to other factors, such as changes in the neural control of muscles with age, which could be especially related to the progressive degeneration of motor neurons, changes in integrity of neuromuscular junctions, denervation of muscle fibers and loss of motor units [5, 6]. Furthermore, it has also been suggested that dynapenia potentially involve disturbances in the central nervous system [7, 8]. In fact, specific brain structures have been associated to muscle tissue and function [9, 10]. For example, reduced gray matter volume in cerebellum has been linked to physical frailty [11] and age-related parietal atrophy is increased in sarcopenic older adults [12]. Moreover, it was suggested that in sarcopenia, arterial stiffness affecting the microcirculation might deteriorate white-matter tracts in the brain [10, 13, 14]. However, a clear difference in structural brain volume is not always observed in sarcopenia [15]. Thus, while some studies highlighted the relationships between muscle health and structural changes in brain, the role of functional brain changes have not been fully explored.

In fact, there is evidence that sarcopenia affects not only motor function, but also involves a decline in cognitive function such that the diagnosis of sarcopenia may constitute a risk factor for incident cognitive decline [16–19]. A clear cut neuropsychological profile associated with sarcopenia has not been identified so far, but different aspects of cognition such as language, memory and executive functions seem affected [17, 20–22].

One paradigm that is often studied in geriatric research on cognitive impairment and its link to motor function in elder participants are dual-task paradigms. Carrying out more than one task at a time is an ability that is crucial in everyday life, and this ability declines with age [23–25]. Moreover, it has extensively been investigated that in the elderly, difficulties to perform a dual-task are related not only to progressive cognitive loss, but also to subsequent adverse events such as falls [26, 27]. Furthermore, recent neuroimaging studies reported that seniors with history of falls show reduced brain activation during performance of a cognitive dual-task [26], suggesting that underlying neural dysfunction may be responsible for altered dual-task performance in fallers. For these reasons, the ability of performing a dual-task might represent also a relevant indicator for elder persons diagnosed with dynapenia, and characterizing the subjacent neural substrates of these changes would provide valuable new insights on this pathology.

However, to our knowledge, no neuroimaging studies have investigated functional changes in brain activity during cognitive tasks in sarcopenic patients thus far. In this study, we therefore probed for any distinctive neural pattern in dynapenia at the level of functional brain processes. To this aim, we used functional Magnetic Resonance Imaging (fMRI) in elderly subjects to test the hypothesis whether dynapenia is associated with changes in brain activity during dual-task conditions close to daily life cognitive demands.

## Methods

### Subjects

For this single-centre cross-sectional study, participants were recruited from a large sample in the context of two clinical randomized controlled studies conducted in Geneva (Switzerland). Participants had to meet the following criteria: age over 65 years, living in the community, Mini-Mental State Examination (MMSE) score of more than 18, and no serious neurological, neuromuscular, or orthopedic condition. Since the majority of the people who agreed to participate were women, only women were included in the final sample. The sample included 59 community-dwelling older women (mean (± SD) age 73.1 years ± 4.9 years). All participants were French speaking, right-handed (determined with the Edinburgh Handedness Inventory), and had normal or corrected-to-normal vision. Ethical approval was given by the institutional review board of the University Hospital of Geneva (protocol 2017–00,437). Written informed consent was obtained from all study participants.

### Clinical scores assessment and analysis

Low muscle strength was defined according to handgrip strength (JAMAR® Plus + Digital hand dynamometer) and/or repeated chair stand (i.e., measure of the time needed to rise five times from a seated position as fast as possible with the arms crossed on the chest) measurements. Participants were subsequently categorized as dynapenic or non-dynapenic according to the EWGSOP2 cut-off points [3]. In addition, for functional physical performances, each participant underwent a functional outcome battery including (i) the Short Physical Performance Battery (SPPB), a composite test that includes assessment of standing balance, 4 m gait speed, and the repeated chair stand test [28], (ii) the Timed “Up & Go” test (TUG) [29], and (iii) the simplified Tinetti test [30, 31]. All patients underwent also a short neuropsychological test battery including the MMSE [32] to assess global intellectual efficiency and the frontal assessment battery (FAB) [33] to assess executive control-related abilities.

### Behavioral task

We designed a novel behavioral dual-task paradigm to investigate motor control performance across different task complexity levels, involving both single-task and multi-task conditions (see supplemental Figure S1). The behavioral paradigm followed the format of a classic dual-task design. Participants were presented with visual stimuli from four different conditions. (i) In the visual baseline condition (B), participants had to watch passively a fixation cross, followed by a screen displaying a circle without any additional visual cue. (ii) In the Motor condition (M), participants were presented with the same circle but with a red visual cue at different locations around the circle, and they were asked to move the joystick in the direction of the red cue as fast as possible. (iii) In the Arithmetic (A) condition, two numbers were presented in the circle, and participants had to perform a mental calculation task and indicate whether a subsequently proposed sum was correct or not. (iv) In the dual-task (D), both tasks (Motor and the Arithmetic) had to be performed simultaneously.

### Functional image acquisition and analysis

All structural and functional brain images were acquired in a 3 T MRI scanner (Siemens TIM Trio, Germany) with a standard 12-channel head coil using a multi-slice echo-planar sequence in single shot for functional images (T2*-weighted; TR/TE = 2000/30 ms, flip angle. 85°, Voxel dimensions = 3 mm isotropic, field of view [FOV] = 192 × 192 mm), and a magnetization prepared rapid acquisition gradient echo sequence for structural images (T1-weighted; TR/TE = 1900/2.27 ms, flip angle = 9°, Voxel dimensions = 1 mm isotropic, Matrix. 256 × 256).

Statistical parametric mapping software (SPM12, fil.ion.ucl.ac.uk/spm/software/spm12) was used for the preprocessing and analysis of the functional brain data. Functional images were realigned and coregistered to the anatomical image. The realigned functional images were spatially normalized to the Montreal Neurological Institute (MNI) stereotactic template brain by using the segmentation procedure implemented in the Computational Anatomy Toolbox (CAT12; neuro.uni-jena.de/cat/). Normalized images were spatially smoothed by using an isotropic Gaussian kernel with a full-width half-maximum of 8 mm.

For the functional image analyses, we implemented a general linear model within SPM where the trials assigned to one of the four task conditions (*baseline *(*B*)*, motor *(*M*), *arithmetic *(*A*), *dual-task *(*D*)) were modelled as separate regressors. Each trial had a duration of 2000 ms. For the motor tasks (i.e. simple and dual conditions), the regressor onset was the time of appearance of the visual cue, while for both arithmetic tasks the regressor onset represented the sum proposition display. All regressors were convolved with the canonical hemodynamic response function (cHRF) implemented in SPM12. Six additional movement realignment parameters were entered as covariates of no-interest. Statistical parametric maps were calculated for each of the four main-effect conditions and were then fed into a second-level (whole brain) flexible factorial analysis, with a factor “condition” with 4 levels (baseline, motor, arithmetic, dual-task), a “group” factor (dynapenic and non-dynapenic), and a “subject” factor. In addition, the variable ‘age’ was added in the analysis as covariate of no-interest.

In a first step, cerebral activations shared by both groups were computed for each of the three networks of interest (i.e. motor, arithmetic, and dual-task), using conjunction analyses. Specifically, brain activity related to each single task was determined by contrasting a given task condition to the baseline condition, namely for the motor network [Dynapenic (M > B) & Non-dynapenic (M > B)], the arithmetic network [Dynapenic (A > B) & Non-dynapenic (A > B)], and the dual-task network [Dynapenic (D > B) & Non-dynapenic (D > B)].

In a second step, between-group analyses were undertaken to assess brain areas differently recruited by the two groups of participants in both the simple motor and arithmetic conditions separately, as well as in the dual-task condition, by computing the t-contrasts ‘dynapenic versus non-dynapenic’ and ‘non-dynapenic versus dynapenic’ for each experimental condition. In addition, for the dual-task, the “cost” of multitasking was compared between the two groups by comparing the contrast of the dual-task versus the sum of the single task between the two groups [Dynapenic (D > M + A) > Non-dynapenic(D > M + A)] and [Non-dynapenic (D > M + A) > dynapenic (D > M + A)], as suggested previously for the analysis of dual-task paradigms [34].

Thirdly, the same between-group analyses were performed with an additional region-of-interest (ROI) approach, where we specifically interrogated brain structures notoriously implicated in motor coordination and execution, namely the bilateral precentral gyrus (PcG), supplementary motor area (SMA), and the basal ganglia including caudate nucleus, putamen and pallidum. This selection of ROIs was driven by our a-priory hypothesis assuming differential functional brain patterns in motor circuits associated with dynapenia. ROIs were anatomically defined by the WFO-pickatlas (http://fmri.wfubmc.edu/software/PickAtlas).

In a subsequent analysis, beta values extracted from the individual peaks in ROI analyses were submitted to mixed ANOVAS, with the within-factor ‘*condition’* and the between-factor ‘*group’*. The rationale for this analysis was to allow inspecting brain activity across conditions for regions identified by one particular contrast.

For all second-level whole-brain and ROI analyses, we report activations with significant p-values (*p* < 0.05) after family-wise error (FWE) correction for multiple comparisons across the whole brain with a cluster size of > 10 contiguous voxels.

### Statistical analysis of clinical scores

Clinical scores were compared between the dynapenic and non-dynapenic groups using independent t-test statistics. In addition, we aimed at identifying how brain activations in specific task conditions varied as a function of clinical scores of interest. To this aim, we extracted the beta values from the individual peak voxels obtained in the between-group analyses and correlated their signal change relative to baseline with the clinical scores. The correlation analyses were performed once with all participants and once with the dynapenic participants only. In these analyses, only the complete scores from the SPPB as well as TUG, Tinetti, MMSE, FAB, and gait speed were entered as regressors. Pearson correlation coefficients were applied for normally distributed clinical scores and Spearman’s rho was administered for non-normally distributed scores. Bonferroni correction was applied for multiple testing.

For statistical analyses with the clinical scores the software R version 4.2.0 was used.

## Results

### EWGSOP2-criteria

Among the 59 participants, 28 were identified as dynapenic, while 31 did not meet the criteria according to the EWGSOP2 cut-off points (non-dynapenic).

### Clinical scores

Results from the cognitive and functional physical assessments are shown in Table 1. Significant differences between the two groups were observed for the SPPB total score, SPPB chair stand score, SPPB chair stand time, and handgrip strength (*p* < 0.05 for all), while there was no difference in cognitive scores (MMSE: *p* = 0.21; FAB: *p* = 0.39) or in the number of falls during the last 12 months (*p* = 0.48). However, the groups differed significantly regarding age (*p* < 0.005) with the dynapenic participants being older (*M* = 75.2 ± 5.0 years) than the non-dynapenic participants (*M* = 71.1 ± 3.9 years). Therefore, the variable age was added as a covariate of no interest in subsequent fMRI analyses.

**Table 1:** Characteristics of participants

	Non-dynapenic	Dynapenic	Total	*p*-value
	*N* = *31*	*N* = *28*	*N* = *59*	
Age (years), mean (SD)	71.1 (3.9)	75.2 (5.0)	73.1 (4.9)	** < .005**
BMI (*Kg/m*^*2*^), mean (SD)	26.8 (4.5)	25.5 (3.2)	26.2 (34.0)	.21
Nb of falls in the past 12 months, mean (SD)	.9 (1.2)	1.2 (2.0)	1.0 (1.6)	.48
FAB (*score*), mean (SD)	16.1 (1.6)	16.4 (1.0)	16.3 (1.4)	.39
MMSE (*score*), mean (SD)	28.0 (2.2)	27.3 (2.2)	27.7 (2.2)	.21
Gait speed (*m/sec*)*,* mean (SD)	1.1 (.2)	1.0 (.2)	1.0 (.2)	.12
Handgrip strength, mean (SD)	21.8 (4.7)	16.8 (7.1)	19.4 (6.4)	** < .005**
SPPB total score (*score*)*,* mean (SD)	10.3 (.9)	9.3 (1.4)	9.8 (1.3)	** < .005**
SPPB chair stand score (score), mean (SD)	2.7 (.6)	2.1 (1.2)	2.5 (.9)	** < .05**
SPPB chair stand time(sec), mean (SD)	12.6 (1.6)	14.8 (3.8)	13.6 (3.0)	** < .005**
SPPB gait score (*score*), mean (SD)	3.9 (.3)	3.6 (.6)	3.8 (.2)	.07
SPPB gait time (sec), mean (SD)	3.9 (.7)	4.3 (1.0)	4.0 (.9)	.07
SPPB balance score (*score*), mean (SD)	3.7 (.7)	3.5 (.7)	3.6 (.7)	.22
Tinetti (*score*), mean (SD)	.3 (.6)	.8 (1.1)	0.5 (.9)	.07
TUG (*sec*), mean (SD)	10.2 (1.5)	11.0 (1.9)	10.6 (1.7)	.11

Abbreviations: body mass index (BMI), mini-mental state examination (MMSE), frontal assessment battery (FAB), short physical performance battery (SPPB), timed Up & Go (TUG)

### fMRI data

#### Networks for motor, arithmetic and dual-task (whole brain analysis)

Brain networks recruited by the different experimental conditions relative to the visual baseline across the whole population were identified with conjunction analyses for both groups (see supplemental Figure S2). For the motor task, increased brain activity was observed in motor circuits including the SMA, pre- and postcentral gyrus, as well as the superior and inferior parietal lobules. For the arithmetic task, a more extended network comprising prefrontal, parietal, and occipital regions was engaged, with peak activations in occipital areas. The overlap of both tasks (depicted in green in upper panel of supplemental Figure S2) included small clusters in supplementary motor area, pre- and postcentral gyrus, as well as the inferior and superior parietal lobules. More critically, the dual-task recruited a widespread network that almost corresponded to the unified sum of both single tasks, with only small deviations. Peak activations were found in SMA, PcG, and superior frontal gyrus but also in middle occipital gyrus and posterior parietal regions.

#### Between-group analyses

**Fig. 1:**
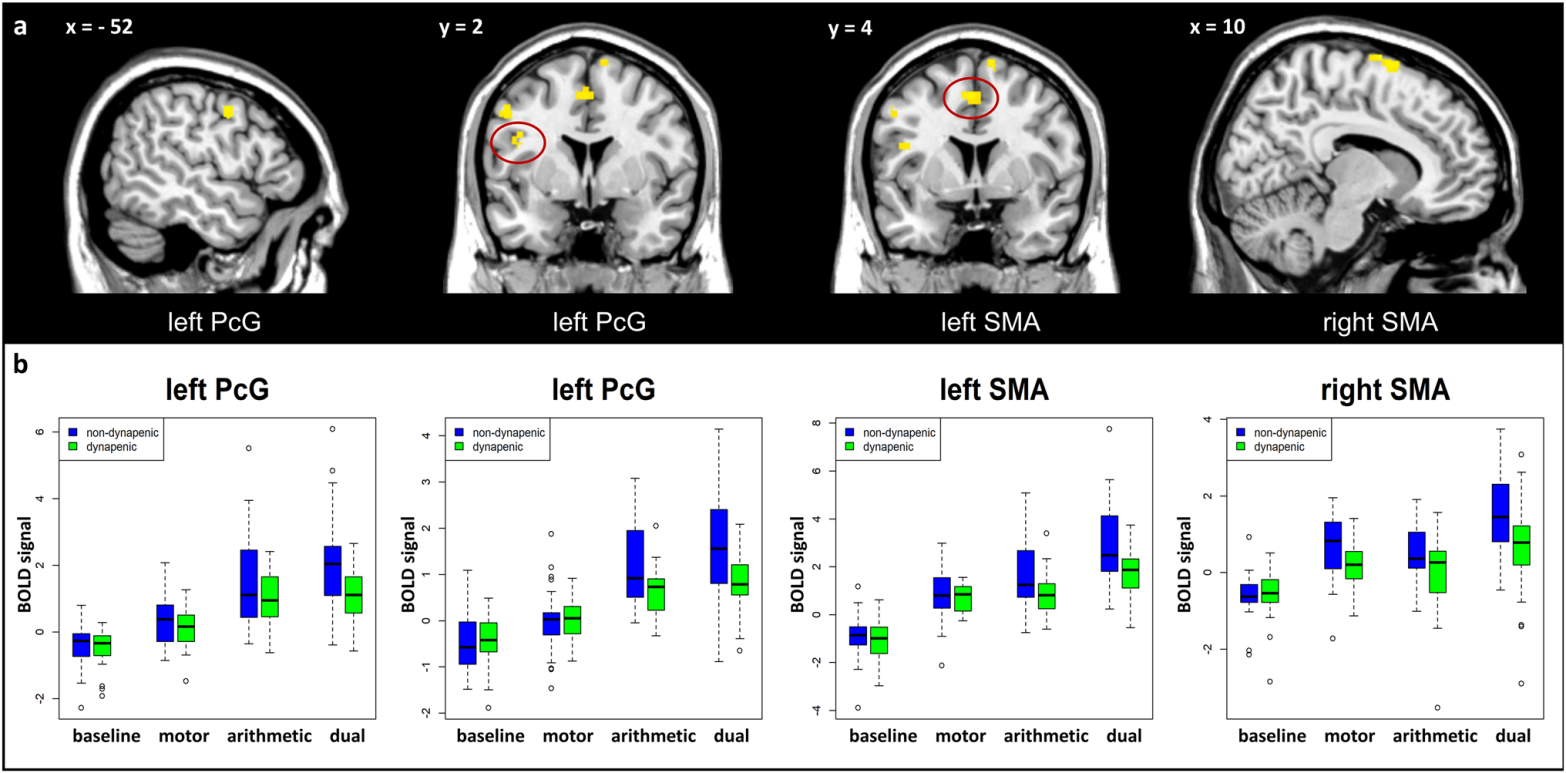
**a** Dynapenia-related brain activity differences during the dual task condition in the contrast ‘non-dynapenic > dynapenic’. *p* < .05, FEW-corrected. **b** Extracted beta values at the peak of the significant clusters for the four conditions. Error bars: CI at 95%. Abbreviations: Precentral gyrus (PcG), supplementary motor area (SMA)

When comparing brain activity between groups in the three experimental conditions, we found no voxels above threshold showing significant increases in the dynapenic group in contrast to the non-dynapenic group, in any of the task conditions. The reverse comparison of the non-dynapenic group relative to the dynapenic group revealed no significant difference for the two single motor or arithmetic tasks. Critically, however, in the dual-task condition, significantly higher activations were found in the non-dynapenic group compared to the dynapenic group, involving four distinct clusters in right middle frontal gyrus (MfG), left PcG, and right SMA (Table 2). Applying a small volume correction with an anatomically defined network of motor areas, comprising PcG, SMA, and striatum (caudate nucleus, putamen, pallidum), we also found four similar clusters, overlapping with the whole brain analysis in left PcG and SMA bilaterally (see Fig. 1a and Table 2).

**Table 2:** Significant clusters for non-dynapenic > dynapenic during the dual-task for the whole brain analysis and small volume correction in ROI-analysis

		Whole brain				ROI-analysis					
AAL location	Side	MNI coordinates			t-value	MNI coordinates			t-value	Mixed ANOVA	
		x	y	z		x	y	z		Interaction *group* x *condition* with Greenhous-Geisser correction	
MfG	R	28	6	48	5.4						
PcG	L	– 52	0	44	5.4	– 52	0	42	5.4	*F*(3,174) = 3.78*	Partial ɳ^2^ = .03
	L	– 44	4	24	4.9	– 42	2	26	4.8	*F*(3,174) = 5.62**	Partial ɳ^2^ = .06
SMA	R	10	0	76	5.0	10	0	76	5.4	*F*(3,174) = 5.62**	Partial ɳ^2^ = .05
	L	– 4	4	52	5.0	– 4	4	52	5.0	*F*(3,174) = 3.72*	Partial ɳ^2^ = .03

t-values and activation peaks showing significantly (*p* < .05, FWE correction for multiple comparisons) higher activation for non-dynapenic participants as opposed to dynapenic participants during the performance of the dual task. Abbreviations: Middle frontal gyrus (MfG), Precentral gyrus (PcG), supplementary motor area (SMA). Mixed ANOVA results on the extracted betas in the ROI-analyses during the dual-task condition with the within-factor ‘*condition’* (baseline, motor, arithmetic, dual) and the between-factor ‘*group’.* ** *p* < .005. * *p* < .05 with Greenhouse–Geisser correction

Neural activity estimates (beta values) across task conditions were extracted from these regional peaks and submitted to mixed ANOVAs to verify the selectivity of group differences. Results showed a significant ‘group x condition’ interaction for all four clusters, supporting a consistent differential increase in non-dynapenic compared to dynapenic individuals during the dual task relative to single tasks (see Fig. 1b and Table 2).

Finally, we also performed a comparison of the “cost” of multitasking between the two groups, by contrasting activations in the dual task relative to the sum of the two single tasks (see methods and [34]). However, this comparison did not reach the threshold of significance (FWE-corrected) in neither of the two directions.

#### Correlation analysis with clinical scores

**Fig. 2:**
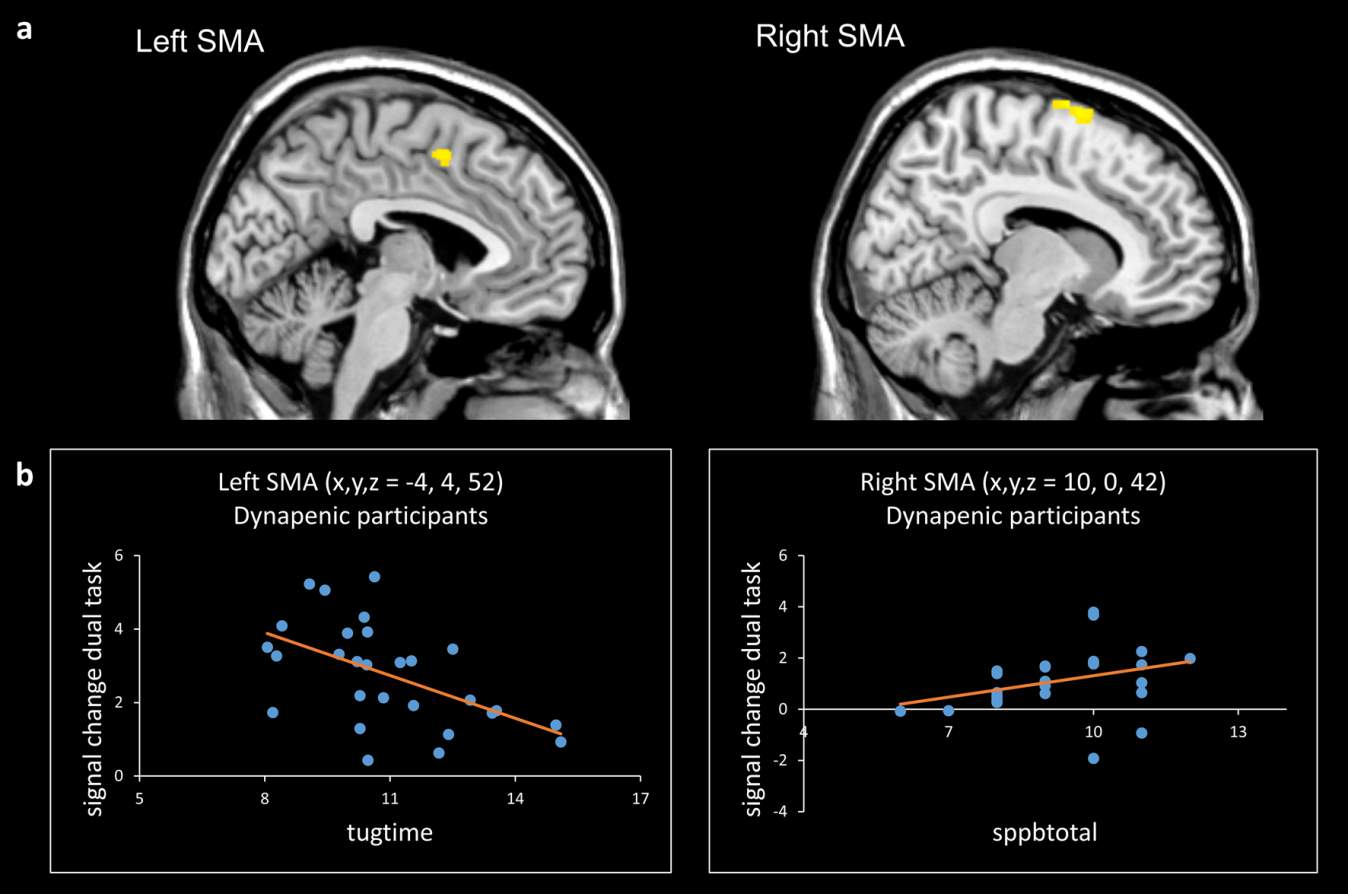
**a** Dynapenia-related brain activity differences during the dual task condition in the contrast ‘non-dynapenic > dynapenic’ in left and right SMA. **b** Scatter plots showing the relation between the BOLD signal change during the dual task in left SMA with the variable *tugtime* (*r* = – .52, *p* = .005) and in right SMA with the variable *sppbtotal* (*r*_*s*_ = .53, *p* = .002). Abbreviations: Precentral gyrus (PcG), supplementary motor area (SMA)

The correlation analyses with clinical scores and brain activation parameters (beta values extracted from relevant ROIs) across all participants revealed a significant positive correlation for the cluster in the right SMA with the SPPB total score (*p* < 0.002) (Table 3). However, given the significant difference between the dynapenic and non-dynapenic groups regarding this variable, such a result could be expected. Performing the same analyses with the dynapenic group only showed that, for the right SMA, a positive correlation with the SPPB total score still remained significant (*r*_*s*_ = – 0.52, *p* = 0.005) (Table 3). In addition, a negative correlation was found in dynapenic participants between the TUG time and activity of the left SMA during the dual-task (*r* = – 0.53, *p* = 0.002) (see Fig. 2).

**Table 3:** Correlation analyses between betas (dual vs baseline) and clinical scores

All participants (N = 59)										
	Pearson correlation						Spearman’s rho			
	TUG	Gait speed	Handgrip	MMSE	FAB		Tinetti	SPPB total	Falls (N)	
left PcG (– 52 0 42)	– .12	.28*	.06	– .03	.04		.08	.26*	.13	
left PcG (– 42 2 26)	– .03	.09	.08	– .07	.00		.05	.19	.04	
left SMA (– 4 4 52)	– .17	.11	– .02	– .12	– .01		.05	.34**	.16	
right SMA (10 0 76)	– .12	.06	.10	– .02	– .03		– .06	.41**	.25	

Dynapenic participants (N = 28)										
	Pearson correlation						Spearman’s rho			
	TUG	Gait speed	Handgrip	MMSE	FAB		Tinetti	SPPB total	Falls (N)	
left PcG (– 52 0 42)	– .44**	.46**	– .09	.29	– .13		.00	.39*	– .06	
left PcG (– 42 2 26)	– .24	.11	– .06	.22	– .14		.03	.12	– .12	
left SMA (– 4 4 52)	– .53**	.20	– .16	.11	.12		– .01	.28	.00	
right SMA (10 0 76)	– .40*	.07	– .08	.01	.08		– .03	.52**	.19	

** *p* < .01, one-tailed. * *p* < .05, one-tailed. Numbers printed in bold indicate significant results according to Bonferroni correction. Abbreviations: Precentral gyrus (PcG), supplementary motor area (SMA)

## Discussion

In this neuroimaging study, we showed for the first time that the syndrome of dynapenia extends also to functional changes in the central nervous system. We demonstrate that low muscle strength diagnosed according to the new criteria of EWGSOP2 is associated with significant decreases in brain activity affecting the prefrontal cortex and motor circuits under a cognitive demanding condition. Specifically, dynapenic older adults show lower recruitment of premotor areas (PcG and SMA) as well as adjacent structures in the prefrontal cortex (MfG) during a dual-task performance, as compared with non-dynapenic older adults. Notably, this functional difference in brain activity between dynapenic and non-dynapenic participants occurred only during the dual-task, whereas the two groups did not differ during the single tasks.

Besides a few shared brain areas activated by both of the single tasks, we were able to identify extended brain networks differentially recruited in both groups for the motor task alone or for the arithmetic task alone (see supplement Figure S2). As expected, the single motor task engaged cortical motor circuits including premotor and parietal areas, while the single arithmetic task activated a large network extending from frontal to parietal and occipital areas. In the critical dual-task condition, very similar brain regions were recruited and mainly comprised a unified summation of both single task effects. These findings accord with previous neuroimaging studies in dual-task paradigms [23, 35–38]. However, in our study we were not able to distinguish any brain activity pattern that would be specific for the dual-task cost, i.e., brain responses unique to the dual condition and independent of the single tasks. However, this classic approach to dual-task paradigms, where the dual-task is contrasted with the sum of the single task to identify unique cost-related effects [34], was not the main focus of the present study. Instead, we were interested in identifying functional differences between dynapenic and non-dynapenic subjects during a demanding cognitive task, and in particular a dual-task condition which represents a situation relevant to everyday life and previously associated with other age-related risk factors [26, 27, 39].

Our comparison of brain activity between the two groups during the dual-task indicated that non-dynapenic subjects recruited more strongly prefrontal (right MfG) as well as motor areas (left PcG and left and right SMA). Dual-task performance has previously been associated with prefrontal cortex functioning [40–42], but also with motor and premotor areas depending on the response modalities and task demands [43, 44]. Given that dynapenia represents a geriatric disease affecting the motor system, our hypothesis was that potential differences in brain activity between dynapenic and non-dynapenic participants would concern primarily motor circuits in the brain. Therefore, we refined our analyses with a particular focus on motor areas including the PcG, SMA, and striatum. Within these ROIs, we could indeed demonstrate that dynapenic and non-dynapenic participants exhibit differential activations during the dual-task condition. In particular, dynapenic participants exhibited lower neural responses in these ROIs relative to the non-dynapenic group, specifically in the dual-task condition, when the motor task and the cognitive task had to be performed at once. This suggests that during high cognitive demands with multi-tasking, dynapenic individuals may present with insufficient upregulation of brain regions that are usually recruited for performing both tasks simultaneously [35, 36, 42].

In previous neuroimaging studies comparing dual-tasking between older and younger, results have typically revealed greater dual-task costs in older adults, with a decrease in behavioral performance in the old compared to the young group [24]. In parallel, it has often been reported that older adults showed higher brain activity compared to younger adults, which has been interpreted as increased structural interference, i.e., higher competition for shared brain regions [23, 35, 45]. However, research on senior fallers and non-fallers has demonstrated that fallers show a decreased brain activity in cognitive tasks [46], and in particular also in dual-task conditions [26]. This converges with our findings, given that dynapenic seniors are more prone to falls compared to healthy controls [47], and therefore partly similar neural mechanisms might be assumed. It should be noted however that in our sample, the reported number of falls within the last 12 months did not differ between groups at the time of the study. Furthermore, hypoactivation in the dual-task condition in dynapenic seniors could also be regarded as a lack of compensatory brain mechanisms. The ‘compensation hypothesis’ in healthy high-performing seniors, i.e. counteracting neurocognitive decline with increased brain activity [48], has also been discussed in patients with mild cognitive impairment to explain their hypoactivation in prefrontal and posterior cortical areas during demanding cognitive tasks [49, 50]. On a neuronal level, reasons for this hypoactivation during cognitive processing with high demands could be due to changes in neurovascular coupling mechanisms in pathologic ageing [51]. In fact, an increased arterial stiffness in sarcopenic seniors might not only lead to deteriorations in white-matter tracts [10, 13, 14], but could also be responsible for reduced neurovascular functioning itself. In addition, it is known that a reduced motor activity, which is the case in dynapenic elderly, is related in turn to a reduced synthesis of brain derived neurotrophic factor (BDNF), which serves as a neuroprotective agent by increasing neurogenesis and preventing neuronal loss [14, 52]. It could thus be assumed that a reduced BDNF in dynapenia could be another underlying mechanism contributing to inefficient neuronal responses, due to physical inactivity [5].

Our results not only highlighted a reduced recruitment in premotor and prefrontal brain regions, but also revealed that this reduction correlated with some clinical performance measures (see Fig. 2b). In particular, we could demonstrate that in the dynapenic group, activity in left SMA during the dual-task correlated negatively with the TUG time, while activity in right SMA correlated positively with the total score of the SPPB. This indicates that the more dynapenic participants showed physical functional impairments, the more their brain activity in SMA was reduced during the dual-task condition. For the single task conditions no such relationships could be observed (see Supplementary Table S1 and S2).

These neuroimaging results might have important implications for interventions for dynapenia. There has been increasing evidence that a dual-task training in older adults may not only have a positive effect on physical performances such as gait and balance [53–55], but also a positive impact on cognition [56–58] and brain function [59]. More research is needed however to confirm that a dual-task training could reduce the symptoms of dynapenia, and to corroborate and extend our findings such that a dual-task training could potentially improve functional brain responses in motor networks and eventually increase muscle strength.

It should be noted, nonetheless, that the present study is not without limitations. First, in our prospectively recruited sample of participants, we observed a significant difference in age between dynapenic and non-dynapenic individuals. In order to control for this issue we added the variable of ‘age’ as covariate in all fMRI analyses. However, we cannot entirely rule out that the slight age difference between groups could have still influenced the results. In a supplement analysis we reanalysed the data by excluding the four oldest women from the dynapenic group and the four youngest women from the non-dynapenic group. This was the smallest number to exclude in order to obtain a non-significant age difference between the groups (*p* < 0.09). This additional analysis revealed rather similar results to the original analysis with the entire sample (see supplement figure S3 and supplement table S3). This could be interpreted such that age did not have a decisive influence on the results.

Second, in the present cohort only women were included. Therefore, the significance of the results is limited to the female population. More research including also sarcopenic men has to be performed in order to extend these results to the male population as well.

Third, unfortunately muscle mass data were not available for the entire cohort. Therefore, the functional brain changes in relation to muscle mass and to the sarcopenia status (as for example, pre-sarcopenia, sarcopenia, and severe sarcopenia according to EWGSOP2 definition) remain to be fully determined.

Fourth, behavioural results from the dual-task could not be fully exploited in our study. Due to technical problems with the joystick in the MRI, behavioural performance during scanning could not be recorded in all participants. Given that these data were incomplete and unbalanced across task conditions, we could not conduct any further analyses of behavioural data. Therefore, behavioural performance regarding reactions times and accuracy can unfortunately not be reported here. This represents a shortcoming of our study, as the results regarding the lower brain responses in dynapenic participants cannot be related to possible impairments in the cognitive task. We can only assume that, similar to studies comparing dual-task performances of senior and young adults, elder persons with a diagnosis of dynapenia would be likely to perform (at least slightly) worse than healthy seniors, due to the reported decrease in cognitive functions in dynapenic seniors [18, 19, 60]. Nonetheless, more research is needed to confirm our neuroimaging results and link the differential brain activity patterns more explicitly with particular behavioural outcomes.

In conclusion, our neuroimaging study is the first to unveil that in older women the syndrome of dynapenia, as defined by the EWGSOP2 criteria, extends to a functional component in the central nervous system. Dynapenic female seniors show reduced brain activity in premotor cortex during the performance of a dual-task, not during single tasks, and this functional reduction in brain activity is associated with worse motor performance in clinical tests. Future clinical research might focus on applications based on our findings in order to design novel therapeutic interventions, and should further investigate if reduced brain activity in motor brain areas could serve as an early sign for adverse events like falls in elderly adults diagnosed with dynapenia.

## Supplementary Information

Below is the link to the electronic supplementary material.

Supplementary file1 (TIF 1637 kb)

Supplementary file2 (TIF 10116 kb)

Supplementary file3 (TIF 8061 kb)

Supplementary file4 (DOCX 20 kb)

## Declarations

### Availability of data and material

The datasets generated during and/or analysed during the current study are not publicly available due to ethical concerns but are available from the corresponding author on reasonable request.
