# Supplementary figures and images for: Functional brain changes in sarcopenia: evidence for differential central neural mechanisms in dynapenic older women
Source: Aging Clin Exp Res. 2023 Apr 8;35(5):1015–25. doi: 10.1007/s40520-023-02391-1 (PMC10149465; doi:10.1007/s40520-023-02391-1)

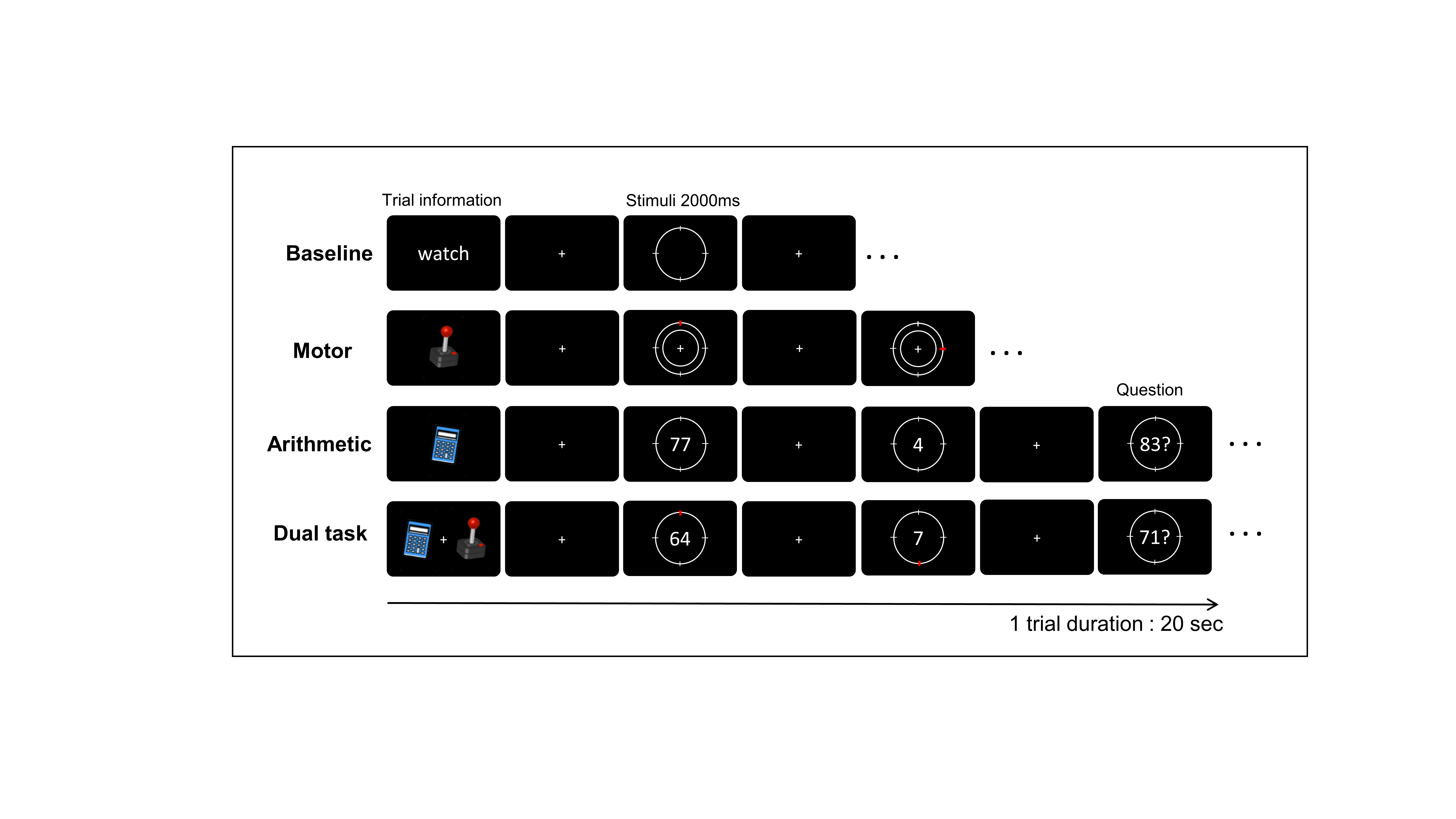

Supplement: Supplementary file 1 — Supplementary file1 (TIF 1637 kb) [file 40520_2023_2391_MOESM1_ESM.tif]

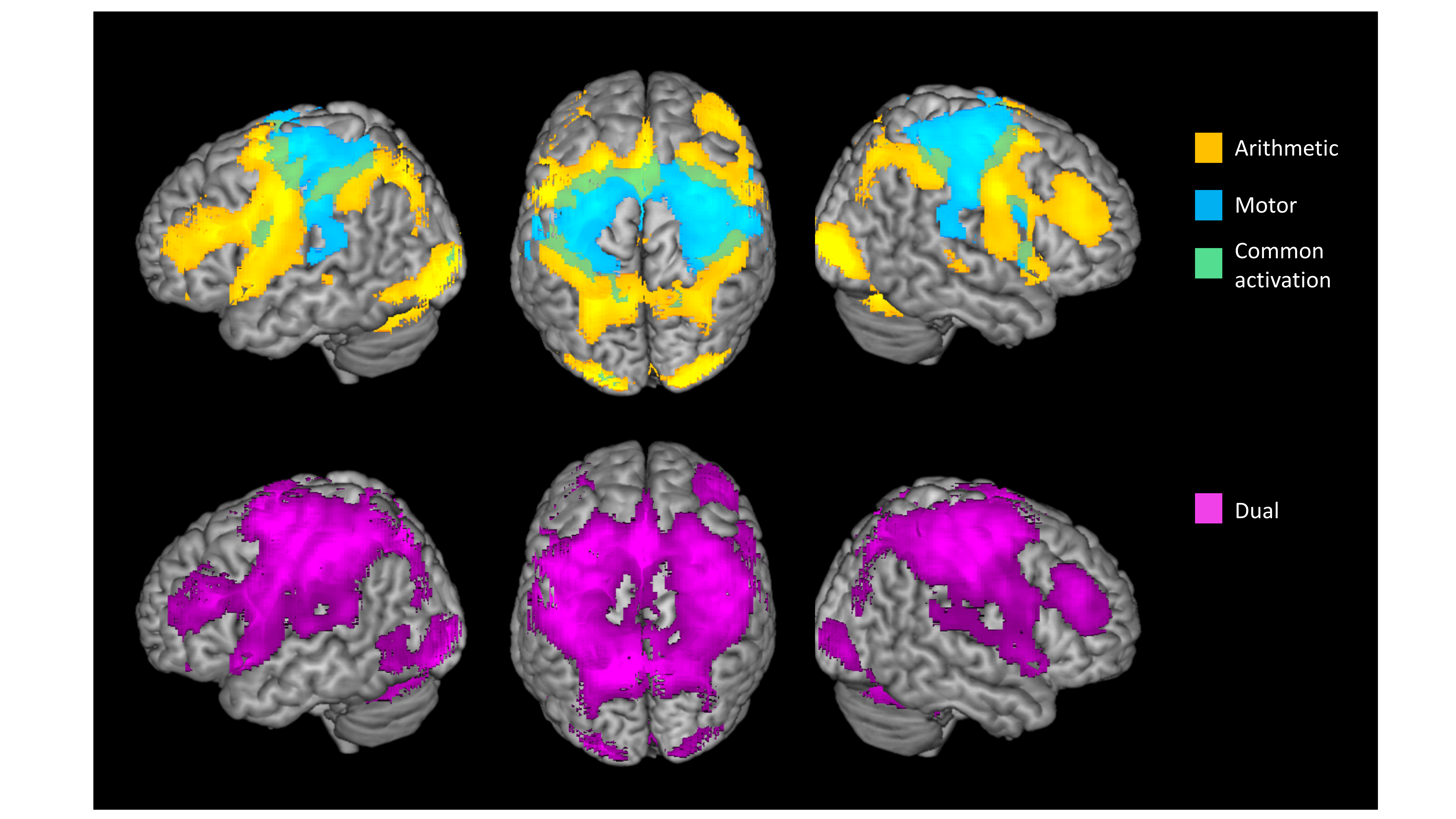

Supplement: Supplementary file 2 — Supplementary file2 (TIF 10116 kb) [file 40520_2023_2391_MOESM2_ESM.tif]

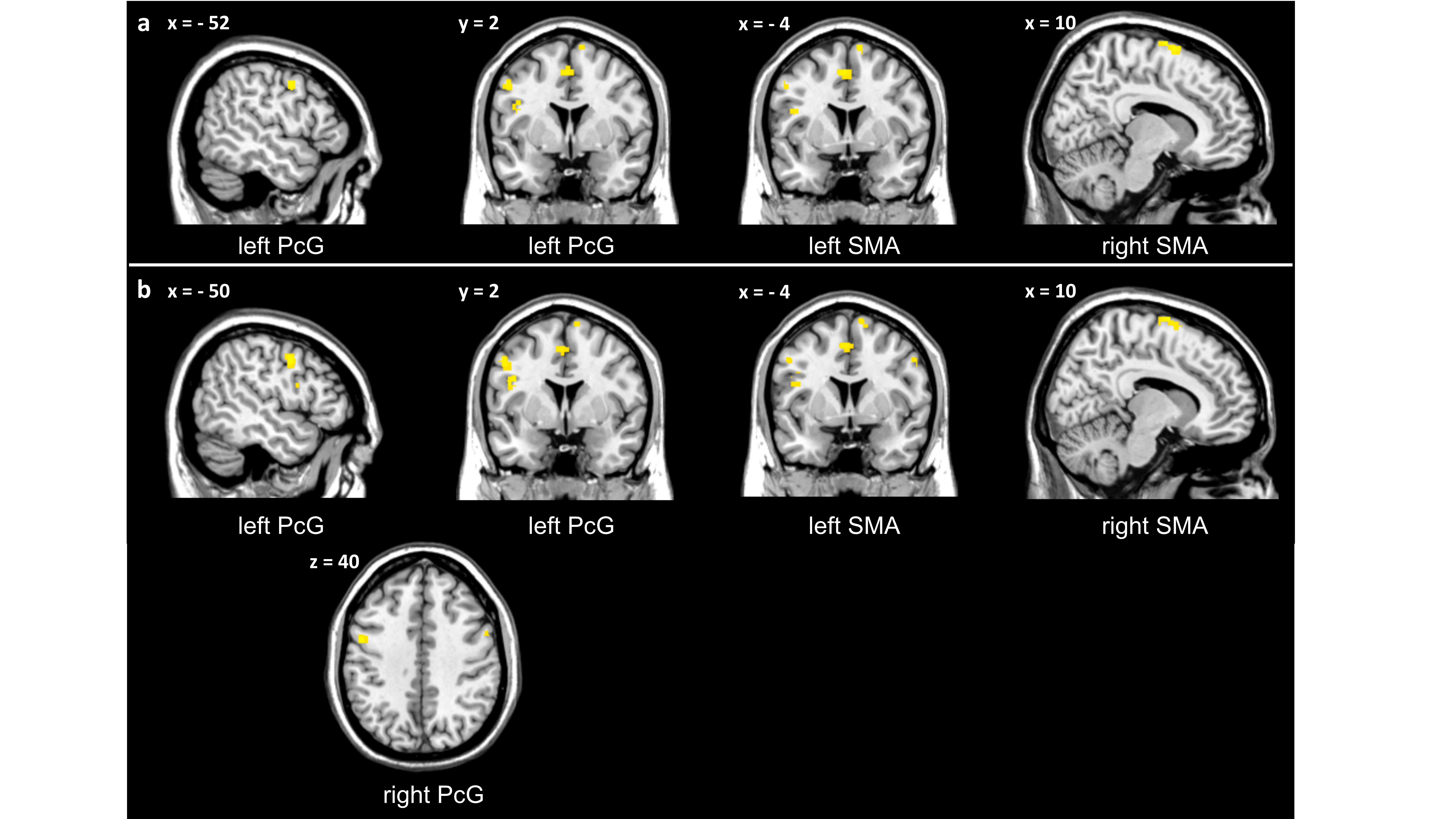

Supplement: Supplementary file 3 — Supplementary file3 (TIF 8061 kb) [file 40520_2023_2391_MOESM3_ESM.tif]
