# Supplementary material for: Functional brain changes in sarcopenia: evidence for differential central neural mechanisms in dynapenic older women
Source: Aging Clin Exp Res. 2023 Apr 8;35(5):1015–25. doi: 10.1007/s40520-023-02391-1 (PMC10149465; doi:10.1007/s40520-023-02391-1)
Supplement: Supplementary file 4 — Supplementary file4 (DOCX 20 kb) [file 40520_2023_2391_MOESM4_ESM.docx]

**Supplement Table S1: Correlation analyses between betas (motor vs baseline) and clinical scores**

|  | | |  |  |  |  |  |  |  |  |  |  |
| --- | --- | --- | --- | --- | --- | --- | --- | --- | --- | --- | --- | --- |
|  | **All participants (N = 59)** | | |  |  |  | **Dynapenic participants (N = 28)** | | | |  |  |
| **Pearson correlation** | TUG | Gait speed | Handgrip | MMSE | FAB |  | TUG | Gait speed | Handgrip | MMSE | FAB |  |
| left PcG(-52 0 42) | .03 | .18 | -.00 | -.05 | -.06 |  | -.27 | .30 | .00 | .12 | -.24 |  |
| left PcG (-42 2 26) | -.01 | .15 | .03 | -.13 | -.06 |  | -.31 | .33* | .08 | .01 | -.15 |  |
| left SMA (-4 4 52) | -.01 | .04 | -.16 | -.15 | -.02 |  | -.23 | .04 | -.11 | .02 | -.22 |  |
| right SMA (10 0 76) | -.17 | .02 | .12 | -.04 | .08 |  | -.00 | .05 | .06 | .02 | -.11 |  |
|  |  |  |  |  |  |  |  |  |  |  |  |  |
|  | **All participants (N = 59)** | | |  |  |  | **Dynapenic participants (N = 28)** | | | |  |  |
| **Spearman's rho** | Tinetti | SPPB total | Falls (N) |  |  |  | Tinetti | SPPB total | Falls (N) |  |  |  |
| left PcG (-52 0 42) | -.05 | .18 | .15 |  |  |  | -.18 | .28 | .00 |  |  |  |
| left PcG (-42 2 26) | -.11 | .17 | .02 |  |  |  | -.05 | .31 | .36 |  |  |  |
| left SMA (-4 4 52) | .09 | .11 | .06 |  |  |  | -.08 | .17 | .28 |  |  |  |
| right SMA (10 0 76) | .04 | .26 | .24 |  |  |  | .19 | .36 | .24 |  |  |  |

** *p* < .01, one-tailed. * *p* < .05, one-tailed. Numbers printed in bold indicate significant results according to Bonferroni correction. Abbreviations: Precentral gyrus (PcG), supplementary motor area (SMA).

**Supplement Table S2: Correlation analyses between betas (arithmetic vs baseline) and clinical scores**

|  | | |  |  |  |  |  |  |  |  |  |  |
| --- | --- | --- | --- | --- | --- | --- | --- | --- | --- | --- | --- | --- |
|  | **All participants (N = 59)** | | |  |  |  | **Dynapenic participants (N = 28)** | | | |  |  |
| **Pearson correlation** | TUG | Gait speed | Handgrip | MMSE | FAB |  | TUG | Gait speed | Handgrip | MMSE | FAB |  |
| left PcG(-52 0 42) | -.10 | .28 | .07 | -.11 | .04 |  | -.21 | .43* | .05 | .12 | -.10 |  |
| left PcG (-42 2 26) | -.06 | .07 | .12 | -.08 | -.07 |  | -.23 | .21 | .07 | .24 | -.04 |  |
| left SMA (-4 4 52) | -.10 | .14 | -.01 | -.15 | -.08 |  | -.41* | .28 | -.19 | .19 | .16 |  |
| right SMA (10 0 76) | -.08 | .03 | .12 | -.08 | -.06 |  | -.32* | .24 | -.01 | .08 | .16 |  |
|  |  |  |  |  |  |  |  |  |  |  |  |  |
|  | **All participants (N = 59)** | | |  |  |  | **Dynapenic participants (N = 28)** | | | |  |  |
| **Spearman's rho** | Tinetti | SPPB total | Falls (N) |  |  |  | Tinetti | SPPB total | Falls (N) |  |  |  |
| left PcG (-52 0 42) | .04 | .08 | .14 |  |  |  | -.05 | .18 | .09 |  |  |  |
| left PcG (-42 2 26) | -.02 | .13 | .02 |  |  |  | -.09 | .10 | -.15 |  |  |  |
| left SMA (-4 4 52) | .01 | .21 | .18 |  |  |  | -.02 | .26 | .00 |  |  |  |
| right SMA (10 0 76) | -.01 | .22 | .21 |  |  |  | .01 | .25 | .15 |  |  |  |

** *p* < .01, one-tailed. * *p* < .05, one-tailed. Numbers printed in bold indicate significant results according to Bonferroni correction. Abbreviations: Precentral gyrus (PcG), supplementary motor area (SMA).

**Supplement Table S3: Additional analysis with homogenized sample regarding age (*n* = 51). Significant clusters for non-dynapenic > dynapenic during the dual-task for the whole brain analysis and small volume correction in ROI-analysis**

|  |  | **Whole brain** | | | | **ROI-analysis** | | | |  |  |
| --- | --- | --- | --- | --- | --- | --- | --- | --- | --- | --- | --- |
| **AAL location** | **Side** | **MNI coordinates** | | | **t-value** | **MNI coordinates** | | | **t-value** | **Mixed ANOVAs** | |
|  |  |  |  |  |  |  |  |  |  | **Interaction *group*  x *condition* with Greenhous-Geisser correction** | |
|  |  | **x** | **y** | **z** |  | **x** | **y** | **z** |  |  |  |
| MfG | R | 28 | 6 | 48 | 5.3 |  |  |  |  |  |  |
|  |  | 40 | 48 | 22 | 5.0 |  |  |  |  |  |  |
| PcG | L | -50 | 0 | 42 | 5.4 | -50 | 0 | 42 | 5.5 | *F*(3,147) = 4.13* | partial ɳ^2^ = .04 |
|  | L | 42 | 2 | 26 | 5.3 | -42 | 2 | 26 | 5.3 | *F*(3,147) = 4.91* | partial ɳ^2^ = .06 |
|  | R |  |  |  |  | 54 | 4 | 40 | 4.5 | *F*(3,147) = 4.00* | partial ɳ^2^ = .04 |
| SMA | R | 10 | 0 | 76 | 5.3 | 10 | 0 | 76 | 5.3 | *F*(3,147) = 5.01** | partial ɳ^2^ = .05 |
|  |  | 12 | -36 | 76 | 5.1 |  |  |  |  |  |  |
|  | L |  |  |  |  | -4 | 4 | 52 | 4.8 | *F*(3,147) = 4.11* | partial ɳ^2^ = .04 |
|  |  |  |  |  |  |  |  |  |  |  |  |

t-values and activation peaks showing significantly (*p* < .05, FWE correction for multiple comparisons) higher activation for non-dynapenic participants as opposed to dynapenic participants during the performance of the dual task. Abbreviations: Middle frontal gyrus (MfG), Precentral gyrus (PcG), supplementary motor area (SMA). Mixed ANOVA results on the extracted betas in the ROI-analyses during the dual-task condition with the within-factor ‘*condition’* (baseline, motor, arithmetic, dual) and the between-factor ‘*group’.* ** *p* < .005. * *p* < .05 with Greenhouse-Geisser correction.
